# Genome Assembly of the Dogface Butterfly *Zerene cesonia*

**DOI:** 10.1093/gbe/evz254

**Published:** 2019-11-22

**Authors:** Luis Rodriguez-Caro, Jennifer Fenner, Caleb Benson, Steven M Van Belleghem, Brian A Counterman

**Affiliations:** 1 Department of Biological Sciences, Mississippi State University; 2 Department of Biology, University of Puerto Rico—Rio Piedras; 3 Division of Biological Sciences, University of Montana, Missoula, MT

**Keywords:** Lepidoptera, de novo assembly, comparative genomics, Hi-Rise assembly

## Abstract

Comparisons of high-quality, reference butterfly, and moth genomes have been instrumental to advancing our understanding of how hybridization, and natural selection drive genomic change during the origin of new species and novel traits. Here, we present a genome assembly of the Southern Dogface butterfly, *Zerene cesonia* (Pieridae) whose brilliant wing colorations have been implicated in developmental plasticity, hybridization, sexual selection, and speciation. We assembled 266,407,278 bp of the *Z. cesonia* genome, which accounts for 98.3% of the estimated 271 Mb genome size. Using a hybrid approach involving Chicago libraries with Hi-Rise assembly and a diploid Meraculous assembly, the final haploid genome was assembled. In the final assembly, nearly all autosomes and the Z chromosome were assembled into single scaffolds. The largest 29 scaffolds accounted for 91.4% of the genome assembly, with the remaining ∼8% distributed among another 247 scaffolds and overall N50 of 9.2 Mb. Tissue-specific RNA-seq informed annotations identified 16,442 protein-coding genes, which included 93.2% of the arthropod Benchmarking Universal Single-Copy Orthologs (BUSCO). The *Z. cesonia* genome assembly had ∼9% identified as repetitive elements, with a transposable element landscape rich in helitrons. Similar to other Lepidoptera genomes, *Z. cesonia* showed a high conservation of chromosomal synteny. The *Z. cesonia* assembly provides a high-quality reference for studies of chromosomal arrangements in the Pierid family, as well as for population, phylo, and functional genomic studies of adaptation and speciation.

## Introduction

Butterflies and moths constitute a monophyletic group of insects characterized by their astonishing diversity in wing color patterns, behaviors, and ecology. Composed of more than 170,000 species, the order Lepidoptera provides a diverse array of phenotypic variation that serves as a model system for studies in genetics, development, ecology, and evolutionary biology (Mavárez et al. 2006; Fujii and Shimada 2007; Bonebrake et al. 2010; Hof et al. 2016; Van Belleghem et al. 2017).

Several aspects of lepidopteran genomes make them distinctly attractive among arthropods and eukaryotes in general: genome sizes are relatively small (∼246–809 Mb), base composition is A–T rich (∼68%) (Triant et al. 2018), structurally they are simple compared with other eukaryotes, and they exhibit a high degree of chromosomal synteny (Papa et al. 2008; Beldade et al. 2009; Yasukochi et al. 2009; Triant et al. 2018). A phylogenetic analysis of Lepidopteran karyotypes revealed that the ancestral number of chromosomes in Lepidoptera was most likely 31, with derived states due to chromosomal fusions documented in Nymphalids (Saura et al. 2013). However, across much of the phylogeny very few chromosomal rearrangements have been documented across the 140 Myr of divergence (Ahola et al. 2014). Comparisons between the genomes of *Bombyx* silk moths and *Heliconius* butterflies confirm that chromosomal organization is broadly conserved between the two lineages (Pringle et al. 2007; Heliconius Genome Consortium 2012), supporting high conservation of synteny across Lepidoptera.

Transposable elements (TE) are abundant in lepidopteran genomes. Lepidoptera TEs have been important sources of genetic tools, such as the piggybac transposon that was initially discovered in cabbage looper moth genomes (Cary et al. 1989), and has now been engineered as a tool for gene-editing in mammalian genomes (Ding et al. 2005; Wilson et al. 2007). TEs have also been important sources of natural variation, as seen in the classic example of industrial melanism in peppered moths, where a TE insertion resulted in darker wing coloration and better camouflage, thereby saving the moth population and providing one of the best modern examples of natural selection in action (Cook and Saccheri 2013; Hof et al. 2016). A recent survey of mobile DNA in arthropods also identified the order Lepidoptera as a hotspot for potential horizontal transfer events, in association with the widespread presence of baculovirus infections, a group of viruses known for their ability to transport host TEs (Reiss et al. 2019). Collectively, the TEs that compose a large amount of lepidopteran genomes offer a remarkable array of opportunities to better understand the evolution of genome architecture and function.

Lepidopteran genomes are constantly being sequenced. However, sequencing efforts have largely concentrated on a few specific clades. As of 2018, there were 48 lepidopteran genome assemblies available, coming from only 8 of the 43 lepidopteran Superfamilies (Triant et al. 2018). Among butterflies, the best represented clade is the family Nymphalidae with more than 20 genomes available, some of which have chromosomal assemblies constructed by pedigree linkage maps, whereas groups like Papilionidae and Pieridae are represented only by a few genomes that range widely in quality. Most of these genomes are accessible through the Lepbase database (www.lepbase.org; last accessed November 30, 2019), a central repository for Lepidoptera genomes that provides an Ensemble genome browser, assembly statistics, and basic sequence analysis tools (Challi et al. 2016).

The southern dogface, *Zerene cesonia*, is a Pierid butterfly distributed across the Americas that exhibits interesting characteristics such as sexually dimorphic development, structural coloration, and developmental plasticity (Fenner 2019). The currently available Pierid genomes are mostly low-coverage draft assemblies, and only six species have genome sequences available (Cong et al. 2016; Shen et al. 2016; Talla et al. 2017). With the aim to generate high-quality genomic resources for the study of *Z. cesonia* and other Pierids, we sequenced the genome of *Z. cesonia* using the Chicago protocol (Putnam et al. 2016) with high-sequencing coverage. We provide RNA-seq based gene annotations and have compared the resulting assembly to representative genomes from other lepidopteran lineages. Our results provide high-quality genomic resources for further understanding the ecology, development, and evolution of Pierid butterflies.

## Materials and Methods

### DNA Sampling and Sequencing

Three female *Z. cesonia* individuals from a colony established at Mississippi State University were frozen in liquid nitrogen 24 h after pupation and sent to the Dovetail Genomics Center. DNA was extracted from two male pupae using Qiagen Genomic-tip DNA isolation protocol. Two Illumina pair-end 150-bp libraries were prepared using the TruSeq DNA PCR-free kit with insert sizes of 550 and 350 bp for DNA shotgun sequencing with HiSeq 2500 and HiSeqX technologies, respectively.

### Genome Assembly

Reads were preprocessed using Trimmomatic (Bolger et al. 2014). First, ILLUMINACLIP was used to remove sequencing adapters. Next all bases with quality scores <20 were removed from the leading and trailing ends of the reads. A sliding window of 13 bp from the end of the read was then used, truncating the read when the average quality dropped <20. After this process, any read shorter than 23 bp was rejected.

Genome size was estimated from *k*-mer frequency method (Guo et al. 2015) and flow cytometry. The *k*-mer distribution with *k* equal to 79 bp best fitted the constrained heterozygous model and was therefore used to estimate the genome size. Genome size was also independently estimated using flow cytometry from DNA isolated from the heads of four *Z. cesonia* individuals and *Drosophila virilis* DNA (330 Mb genome size) as reference.

A preliminary genome assembly was generated using Meraculous (Chapman et al. 2011) for contig reconstruction, and the Chicago protocol for scaffolding. The Chicago protocol generates proximity ligation libraries using reconstituted chromatin as a substrate and then creates scaffolds using the HighRise (HiRiSE) software (Putnam et al. 2016). Both procedures were performed by Dovetail genomics. This initial preliminary assembly, named Z_cesonia_v-0.1, was constructed with a single DNA library (550 bp insert size) using Meraculous in diploid mode 1. Because this strategy failed to capture the sex (Z) chromosome, we generated a second assembly named Z_cesonia_v-0.2 with increased coverage and using Meraculous in diploid mode 2 to increase the probability of capturing all chromosomes. As Z_cesonia_v-0.2 was a diploid assembly, we used the Haplomerger pipeline (Huang et al. 2012) to assemble a single reference haplome for Z_cesonia_v-0.2. To confirm that diploid regions were successfully merged by Haplomerger, we aligned Z_cesonia_v-0.2 with Z_cesonia_v-0.1 using MUMmer (Marçais et al. 2018) and used a custom python script was designed to remove duplicate portions of scaffolds that Haplomerger failed to detect. The last step was to order and orient scaffolds from Z_cesonia_v-0.2 using the Chicago library preparation and Hi-Rise assembly information of Z_cesonia_v-0.1. For this, we used RaGOO (Alonge et al. 2019) with zerene_cesonia_0.1 as a reference. A detailed report describing the procedures used to produce zerene_cesonia_v-1.0, including the scripts and coordinates used for duplicate removal can be found in the GitHub repository for this project (https://github.com/LF-Rodriguez/Z_cesonia_genome_assembly_2019/tree/master/supplemental; last accessed November 30, 2019).

The mitochondrial genome was assembled using the libraries above and NOVOplasty2.7.2 (Dierckxsens et al. 2017). The Novoplasty assembler was run using recommended parameters from the documentation, with sequencing adapters trimmed from reads, and a *Z. cesonia* partial CDS of the cytochrome oxidase 1 subunit (GenBank accession no. GU164697) for the input seed sequence.

### Genome Annotation

Repetitive elements (REs) were masked with RepeatMasker (www.repeatmasker.org; last accessed November 30, 2019) using a customized library containing repeats from all hexapoda including all annotated repeats from *Heliconius* butterflies updated in 2007. We used the Maker-2 pipeline (Holt and Yandell 2011) to annotate the genome, using a transcriptome of *Z. cesonia* assembled de novo from wing disc, thorax, and head tissues (SRA bioProject ID: PRJNA587792) as evidence for mRNA and exon boundaries.

To explore chromosomal synteny, we performed a MUMmer alignment of the 29 largest scaffolds of Z_cesonia_v-1.0 (91.4% of the assembly) to the 20 autosomes of *H.erato* (v.1.0), which contains chromosome information inferred from pedigree linkage maps. The scripts and references used for genome alignments and chromosome assignment are available at the GitHub repository for this project (https://github.com/LF-Rodriguez/Z_cesonia_genome_assembly_2019; last accessed November 30, 2019). Protein-coding genes in the mitochondrial genome assembly were identified and annotated using sequence similarity with *Colias erate* (GenBank accession no. NC_027253).

## Results and Discussion

### DNA Sampling and Sequencing

We obtained a total of 191,008,162 reads from the first DNA library (HiSeq 2500) of which 91.8% passed the trimmomatic filter with a final average length of 142.4 bp. The second DNA library (Hi-SeqX), generated 387,729,917 reads of which 97.9% passed the trimmomatic filters with final average length of 143.2 bp.

### Genome Assembly

Generating an accurate estimate of the genome size of *Z. cesonia* allowed us to evaluate the completeness of the assemblies. The *k*-mer distribution analysis estimated a genome size of 271 Mb using a *k* of 79 which best fitted the distribution of the heterozygous model. Using flow cytometry and *Drosophila virilis* as a reference, the *Z. cesonia* genome was estimated to be 303 Mb, with a SD of 6 Mb. For characterizing assembly metrics, we used the 271 Mb *k*-mer genome size estimate.

**Fig. 1.**
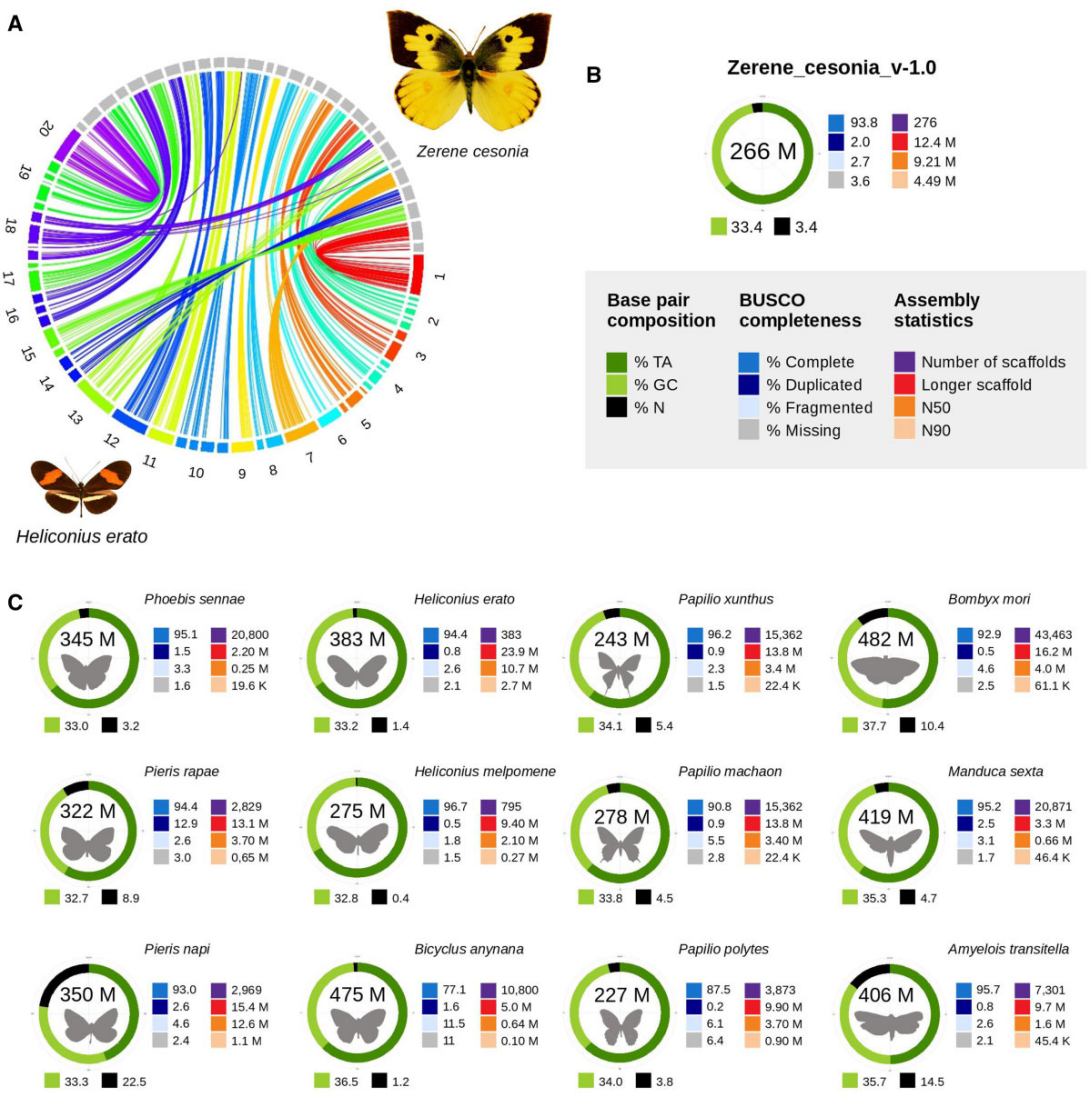
—*Zerene cesonia* genome assembly in comparison to other lepidopterans. (*A*) Synteny conservation between *Z. cesonia* and *Heliconius erato* genomes. (*B*) Genome assembly and composition for *Z. cesonia* assemblies. (*C*) Graphical comparison of genome assemblies across butterflies and moths.

The preliminary assembly, Z_cesonia_v-0.1, was constructed with ∼195× coverage into large scaffolds, most of which were near the expected chromosomal sizes, and covered a total of 229,153,833 bases (84.5% of the estimated genome size). This assembly conducted by Dovetail benefitted from the Chicago library prep and Hi-Rise assembly, but had insufficient coverage to assemble the Z chromosome, and a relatively large number of ambiguous bases (i.e., *N*) inserted in the genome (12.1%). The second assembly, Z_cesonia_v-0.2, was constructed with increased coverage (∼322×) using a second male individual, and the Meraculous assembler in diploid mode 2. This resulted in a diploid genome assembly of ∼516.4 Mb, with most autosome sequences present twice as expected from the Meraculous diploid mode 2 configuration. This approach allowed us to assemble the full Z chromosome in a single scaffold (12.4 Mb). Merging the diploid Z_cesonia_v-0.2 assembly into a haplome resulted in a final haploid assembly (Z_cesonia_v-1.0) of size 266,407,278 bp, which is 98.2% of the expected genome size (271 Mb), represented by a total of 276 scaffolds with an N50 of 9.2 Mb (fig. 1). Notably, 91.4% of the genome assembly is contained in the 29 largest scaffolds.

The assembly of *H.erato’s* genome is a valuable reference for butterfly genomics because of its chromosomal assemblies that were constructed empirically using pedigree-based linkage maps (Van Belleghem et al. 2017). Assuming high-synteny conservation between Pierid and Nymphalid genomes, we mapped the genome of *Z. cesonia* to the genome of *H. erato* to determine the identity of the scaffolds. According to previous studies on lepidopteran genomes (Maeki 1960; Saura et al. 2013), the genome of *Z. cesonia* has 31 chromosomes (29 autosomes). We found that the 29 largest scaffolds of the assembly largely show homology to only one or two chromosomes in *H. erato* (fig. 1*A*). This suggests that these 29 scaffolds likely reflect near full assemblies of 28 autosomes and the Z chromosome. This also suggests that, similar to other major clades of Lepidoptera, Pierid genomes also exhibit high conservation of chromosomal synteny.

The *Z. cesonia* mitochondrial genome assembled into a single contiguous sequence of 15,138 bp with a GC-content of ∼19.2%, and the positions of 13 protein-coding genes identified.

### Genome Annotation

Extensive research in butterfly genomics has generated a thorough repertoire of annotated genomic features including coding sequences and a curated library of REs for butterflies (Challi et al. 2016). Taking advantage of those resources, we used Maker (Holt and Yandell 2011) to annotate the genome and RepeatMasker (Tarailo-Graovac and Chen 2009) to identify and mask REs.

**Fig. 2.**
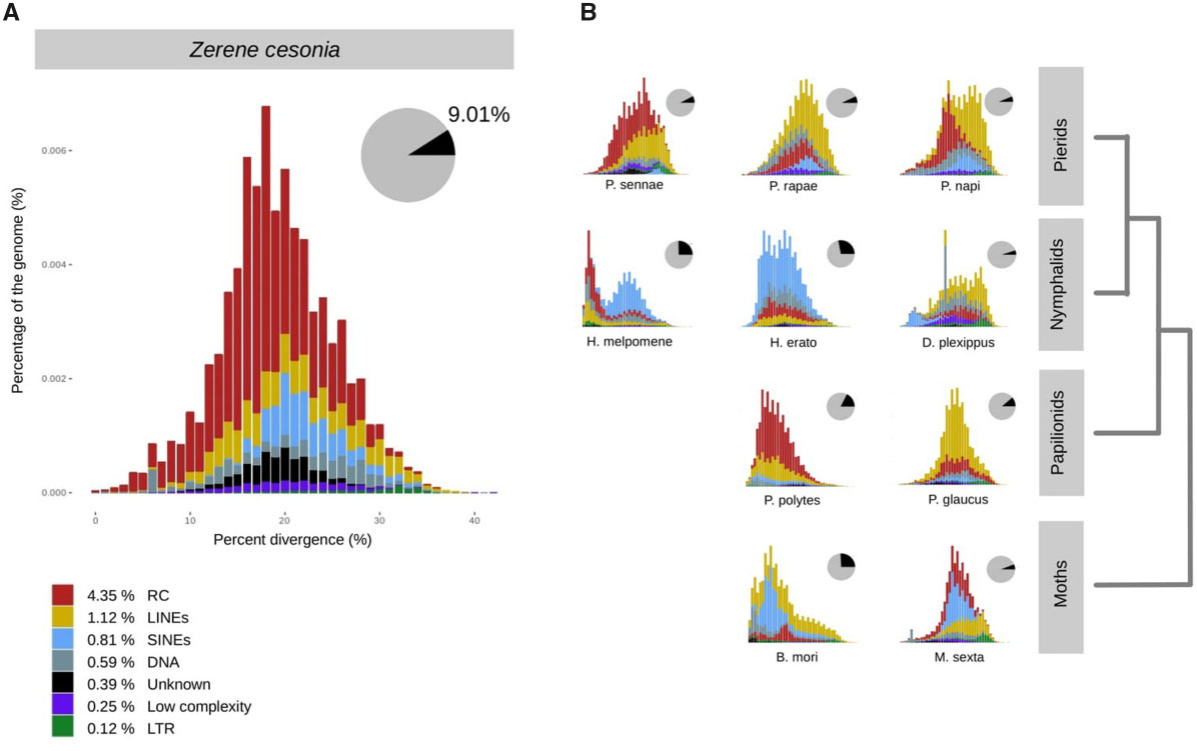
—Comparison of repetitive element (RE) abundance in *Zerene cesonia* and other lepidopterans. (*A*) Bar plot of RE types abundance versus their divergence. Divergence is estimated as the percentage of divergent sites on the RepeatMasker hits. The pie chart shows the portion of the genome containing REs in black. (*B*) Bar plots of abundance versus divergence of REs in other lepidopterans. All bar plots range from 0% to 40% in the *x* axis.

We identified 16,442 genes with an average gene span of 5,757 bp and found that the 9.01% of the genome is composed of RE, most of which are helitrons. This is a small portion of RE compared with butterflies like *H.erato* (27.95%; Van Belleghem et al. 2017), *Heliconius melpomene* (25.36%; Ray et al. 2019), the silkworm moth, *Bombyx mori* (35.4%; Osanai-Futahashi et al. 2008), and other pierid butterflies (*Phoebis sennae*—22.7%, and *Pieris rapae*—17.2%; Shen et al. 2016; Talla et al. 2017) (fig. 2*B*). Divergence plots of TE families shows clear differences in TE content among the different Lepidoptera lineages. Divergence of TE families was measured as the percentage of divergent bases for each genomic copy compared with the consensus sequence generated from RepeatMasker.

Although a more accurate RE annotation and standardized divergence metrics are required to make inferences about the history and evolution of REs in the genome (Platt et al. 2016), raw percent divergence is informative to identify overall patterns of RE activity across lineages. The distributions of RE divergence in *Z. cesonia* reflects high divergence of all RE types (peak ∼20%) which suggests inactivity of REs in the recent past. This contrasts with the patterns observed in the genus *H.melpomene* (fig. 2B), which is known to have experienced recent transposon activity (Lavoie et al. 2013) and substantial genome diversification due to TE activity (Ray et al. 2019). When comparing the TE landscape among lepidopteran genomes, two patterns can be identified. First, LTR divergence peak is close to 20% in most groups included, suggesting that their activity ceased before the split between moths and butterflies, which is consistent with the findings of previous studies of RE activity in Lepidoptera (Lavoie et al. 2013; Talla et al. 2017; Reiss et al. 2019). Secondly, helitrons and LINEs are the most abundant type of REs among Pierid butterflies, but overall the species included here show low abundance of REs (range: 6.17–22.7%) and no signs of RE activity in their genomes in the recent past. Additionally, *P. sennae* and *Z. cesonia* show a relatively large abundance of undescribed RE’s compared with other lepidopterans, which reflects an incomplete characterization of Pierid-specific RE’s.

Together, these results provide a comprehensive summary of the composition and architecture of the genome of *Z. cesonia.* The assembly here presented covered 98.2% of the genome with chromosome sized scaffolds and provide an initial characterization of the TE landscape of *Z. cesonia* and other lepidopterans. 

## Supplementary Material

Supplementary data are available at *Genome Biology and Evolution* online.

## Supplementary Material

evz254_Supplementary_Data

## Notes

**Data deposition:** RNA-seq data used for annotation are deposited in the SRA database. Accession bioProject ID: PRJNA587792.
