# Supplementary material for: Genome Assembly of the Dogface Butterfly Zerene cesonia
Source: Genome Biol Evol. 2019 Nov 22;12(1):3580–5. doi: 10.1093/gbe/evz254 (PMC6944212; doi:10.1093/gbe/evz254)
Supplement: evz254_Supplementary_Data [file evz254_supplementary_data.zip › ZcesGenome_SF1.doc]

**Figure S1.** Schematic representation of the strategy used for generating a high quality assembly of the genome of *Zerene cesonia*. The initial preliminary assembly (v-0.1) generated long, but highly gaped scaffolds (black bands represent gaps) and failed to capture sex chromosomes. The diploid assembly (v-0.2) captured all chromosomes and had less gaps, but remained fragmented. Using Haplomerger, v-0.1 was merged into a haplome and using RaGOO, scaffolds were oriented and assembled into chromosome sized super scaffolds using v-0.1 as a reference.
