# Supplementary material for: Genome Assembly of the Dogface Butterfly Zerene cesonia
Source: Genome Biol Evol. 2019 Nov 22;12(1):3580–5. doi: 10.1093/gbe/evz254 (PMC6944212; doi:10.1093/gbe/evz254)
Supplement: evz254_Supplementary_Data [file evz254_supplementary_data.zip › ZcesGenome_ST1.doc]

|  | | Pierids | | | Nimphalids | | | Papilionids | | Moths | |
| --- | --- | --- | --- | --- | --- | --- | --- | --- | --- | --- | --- |
|  | **Feature** | Zces | Pse | Pra | Her | Ban | Dpl | Ppo | Pxu | Bmo | Mse |
| Structure | Assembly size (Mbp) | 266 | 345 | 322 | 383 | 475 | 249 | 227 | 244 | 482 | 419 |
|  | N content (%) | 3.4 | 3.2 | 8.9 | 1.4 | 1.3 | 2.7 | 3.8 | 2.4 | 10.4 | 4.7 |
|  | No. scaffolds | 276 | 20,800 | 2829 | 196 | 10,800 | 5,397 | 3,874 | 5,572 | 43,462 | 20,871 |
|  | Longest scaffold (Mbp) | 12.4 | 2.2 | 13.1 | 23.9 | 5 | 6.2 | 9.9 | 16.3 | 16.2 | 3.3 |
|  | GC content (%) | 33.4 | 33.0 | 32.7 | 33.2 | 36.5 | 31.6 | 34.0 | 33.8 | 37.7 | 35.3 |
|  | N50 (Mbp) | 9.21 | 0.256 | 3.7 | 10.7 | 0.64 | 0.72 | 3.7 | 6.2 | 4.0 | 0.66 |
|  | N90 (Mbp) | 4.49 | 0.02 | 0.65 | 2.7 | 0.09 | 0.16 | 0.93 | 0.53 | 0.06 | 0.05 |
| BUSCO | Complete (%) | 93.77 | 93.6 | 94.4 | 94.5 | 75.5 | 94.7 | 87.3 | 95.3 | 92.4 | 92.7 |
|  | Duplicated (%) | 2.0 | 1.5 | 12.9 | 0.8 | 1.6 | 1.9 | 0.2 | 0.9 | 0.5 | 2.5 |
|  | Fragmented (%) | 2.7 | 3.3 | 2.6 | 2.6 | 11.5 | 2.3 | 6.1 | 2.3 | 4.6 | 3.1 |
|  | Missing (%) | 3.6 | 1.6 | 3 | 2.1 | 11.4 | 1.1 | 6.4 | 1.5 | 2.5 | 1.7 |

**Table S1.** Quality measures of the genome assembly of Zerene cesonia compared to other lepidopterans. Zces: Zerene_cesonia_v-1.0, Pse: *Phoebis sennae*, Pra: *Pieris rapae*, Her: *Heliconius erato*, Ban: *Bicyclus anynana*, Dpl = *Danaus plexippus*, Ppo: *Papilio polytes*, Pxu: *Papilio xuthus*, Bmo: *Bombyx mori*, Mse: *Manduca sexta*.
